# Supplementary material for: Factors Related to Plasma Homocysteine Concentration in Young Adults: A Retrospective Study Based on Checkup Populations
Source: J Clin Med. 2023 Feb 19;12(4):1656. doi: 10.3390/jcm12041656 (PMC9967549; doi:10.3390/jcm12041656)
Supplement: Supplementary file 1 [file jcm-12-01656-s001.zip › jcm-2103370-supplementary.pdf]

Table S1 Correlation analysis between Hcy level and each variable in each age group in young males

age group	Variables	r	*p* value
20-24Y	BMI	-0.139	0.312
	WHR	-0.063	0.650
	ALT	-0.109	0.427
	AST	-0.137	0.319
	CREA	0.006	0.962
	UA	-0.274	0.043*
	TG	-0.228	0.094
	TC	-0.129	0.349
	LDL-C	-0.075	0.585
	HDL-C	0.065	0.637
	Glu	-0.010	0.940
25-29Y	BMI	0.100	0.077
	WHR	0.012	0.835
	ALT	-0.015	0.786
	AST	0.027	0.635
	CREA	0.065	0.251
	UA	-0.035	0.539
	TG	0.014	0.801
	TC	-0.076	0.177
	LDL-C	-0.080	0.154
	HDL-C	0.004	0.938
	Glu	-0.046	0.416
30-34Y	BMI	0.030	0.504
	WHR	-0.003	0.945
	ALT	0.014	0.751
	AST	0.004	0.921
	CREA	0.022	0.624
	UA	0.052	0.241
	TG	0.005	0.915
	TC	-0.032	0.465
	LDL-C	-0.050	0.260
	HDL-C	0.046	0.304
	Glu	-0.053	0.235
35-39Y	BMI	0.079	0.133
	WHR	0.005	0.921
	ALT	-0.002	0.968
	AST	0.004	0.941
	CREA	0.084	0.110
	UA	0.098	0.061
	TG	-0.012	0.816
	TC	-0.057	0.275
	LDL-C	-0.030	0.569
	HDL-C	-0.108	0.039*
	Glu	-0.041	0.438

Notes: BMI, body mass index; WHR, waist-to-hip ratio; ALT, alanine aminotransferase; AST, aspartate aminotransferase; CREA, creatinine; UA, uric acid; TG, triglyceride; TC, total cholesterol; LDL-C, low-density lipoprotein cholesterol; HDL-C, high-density lipoprotein cholesterol; Glu, glucose. Y, years. *, $p<0.05$.

Table S2 Correlation analysis between Hcy level and each variable in each age group in young females

age group	Variables	r	*p* value
20-24Y	BMI	-0.032	0.794
	WHR	0.226	0.059
	ALT	-0.094	0.439
	AST	0.051	0.675
	CREA	0.102	0.403
	UA	0.072	0.556
	TG	0.042	0.729
	TC	0.148	0.222
	LDL-C	0.019	0.878
	HDL-C	0.328	0.006**
	Glu	-0.051	0.673
25-29Y	BMI	0.035	0.511
	WHR	-0.014	0.798
	ALT	-0.070	0.184
	AST	0.033	0.538
	CREA	0.195	＜0.001**
	UA	0.143	＜0.001**
	TG	-0.037	0.483
	TC	0.009	0.861
	LDL-C	-0.017	0.743
	HDL-C	0.011	0.838
	Glu	-0.045	0.398
30-34Y	BMI	-0.022	0.648
	WHR	-0.067	0.165
	ALT	-0.032	0.516
	AST	0.056	0.252
	CREA	0.169	＜0.001**
	UA	0.035	0.473
	TG	-0.056	0.250
	TC	-0.034	0.485
	LDL-C	-0.019	0.701
	HDL-C	-0.037	0.450
	Glu	-0.044	0.362
35-39Y	BMI	0.057	0.293
	WHR	-0.013	0.807
	ALT	0.142	0.009**
	AST	0.213	＜0.001**
	CREA	0.168	0.002**
	UA	0.094	0.085
	TG	0.106	0.052
	TC	0.020	0.710
	LDL-C	-0.003	0.955
	HDL-C	-0.016	0.763
	Glu	-0.035	0.524

Notes: BMI, body mass index; WHR, waist-to-hip ratio; ALT, alanine aminotransferase; AST, aspartate aminotransferase; CREA, creatinine; UA, uric acid; TG, triglyceride; TC, total cholesterol; LDL-C, low-density lipoprotein cholesterol; HDL-C, high-density lipoprotein cholesterol; Glu, glucose. Y, years. **, $p<0.01$.
